# *Cystoisospora* Species Insights From Development *in vitro*

**DOI:** 10.3389/fvets.2018.00335

**Published:** 2019-01-09

**Authors:** David S. Lindsay

**Affiliations:** Zoonotic Protozoal Diseases Laboratory, Department of Biomedical Sciences and Pathobiology, Center for One Health Research, Virginia Maryland College of Veterinary Medicine, Virginia Tech, Blacksburg, VA, United States

**Keywords:** cell culture, *Cystoisospora belli*, *Cystoisospora canis*, *Cystoisospora felis*, *Cystoisospora rivolta*, *Cystoisospora suis*, endodyogeny, tissue cyst

The Genus *Cystoisospora* was created in 1977 (1) to account for the recently described facultative two-host life cycles observed occurring in feline and canine *Isospora* species (2, 3). The justification for the creation of the Genus *Cystoisospora* and placement of mammalian *Isospora* species in it was based on morphological, biological, and genetic differences between *Isospora* species from non-mammalian hosts and mammalian hosts (4–6). The orally infectious tissue cyst stage present in the intermediate host contains a single asexual stage and is referred to as a monozoic tissue cyst (MZTC) because it contains a single organism within a parasitophorous vacuole surrounded by a tissue cyst wall (7–9). The stage is classified as a zoite because it has all the ultrastructural features found in sporozoites, tachyzoites, and bradyzoites of other Sarcocystidae. Most notably the zoite contains a crystalloid body observed in sporozoites of *Cystoisospora* species (10). Crystalloid bodies are spherical accumulations of granular cytoplasmic inclusions similar to beta-glycogen particles. They are in the same location of the sporozoite as are refractile bodies of *Eimeria* species sporozoites excysted from oocysts (11) and occasionally early generation merozoites of *Eimeria* species (12). Crystalloid bodies and refractile bodies are believed to serve as sites of energy storage to maintain the dormant sporozoites until they are ingested by a host and become metabolically active. The presence of crystalloid bodies in sporozoites in oocysts and zoites in MZTC of *Cystoisospora* is now recognized as a feature useful in their identification (6).

It is not known if zoites originate from oocysts that have excysted and represent sporozoites that migrate to extra-intestinal sites without developing or if they represent a post divisional stage, such as a tachyzoite, bradyzoite, or merozoite. This remains one of the most important questions concerning the biology of this group and has important implications in the management of *C. belli* infections in immune compromised patients. However, the tremendous number of MZTC each containing a single zoite observed in tissues of immune suppressed patients with *C. belli* infections indicates that zoites in MZTC represent an asexual stage that has been produced by multiplication either by endodyogeny or another form of merogony and has migrated to its location of encystation and became a MZTC (13).

Pioneering studies on the development of *Cystoisospora* species in cell cultures were conducted by Dr. Ron Fayer at the United States Department of Agriculture in Beltsville, Maryland, from 1972 to 1974 (14–16). These studies demonstrated that sporozoites excysted from oocysts of *C. rivolta* and *C. felis* collected from cats, and sporozoites of *C. canis* from dogs, entered cultured cells and divided repeatedly by endodyogeny. They provided a foundation for future studies of the medically important *C. belli* from humans and economically important *C. suis* in young swine.

*Cystoisospora rivolta* developed in feline kidney cells, embryonic bovine kidney cells, and Madin-Darby canine kidney (MDCK) cells (14). Development was delayed by 24 h and was minimal in in MDCK cells. *Cystoisospora felis* developed in embryonic intestine, esophageal epithelium, amnion, lung, and Hela cells from humans, kidney cells from chickens, embryonic tracheal cells from bovines, and MDCK cells from dogs but cells from cats were not examined (16). Development by *C. felis* was similar in all these cell types.

Development of sporozoites of *C. canis* was examined in MDCK cells and primary cells from embryonic canine kidneys, and embryonic canine intestine as well as embryonic bovine trachea and kidney (15). Development by endodyogeny occurred in all host cell types 3–4 days after inoculation. A study of development of *C. canis* sporozoites using bovine turbinate and African green monkey kidney (CV-1) cells provided remarkedly different findings of no asexual multiplication but rather MZTC formation in both host cell types (17). Examination of these stages using transmission electron microscopy confirmed they were MZTC and that they were similar to *C. belli* MZTC in the tissues of humans (13, 18), *C. canis* in mice (9), *C. ohioensis* in mice (8) and *C. felis* in mice (19). The organisms in these MZTC all contain crystalloid bodies when viewed using transmission electron microscopy. An in-depth study was conducted on the biology of MZTC of *C. canis* using two canine, four human, one monkey, and one bovine cell lines (20). No asexual multiplication occurred in any cell line however MZTC were produced in all cell lines. It is important to note that MDCK cells were used by both Fayer and Mahrt in their study (15) and Houk and Lindsay in their study (20) with dramatically different results. When host cells containing MZTC were exposed to excystation solution (0.75% sodium taurocholic acid and 0.25% trypsin solution) the sporozoites became motile and exited the MZTC and were able to infect new host cells and produce MZTC again. The same events occurred, except the tissue cyst wall was dissolved by the acid-pepsin solution, when MZTC were exposed to acid-pepsin solution suggesting survival through the stomach and oral infectiousness of this stage (20). Additional research is needed to account for these different findings between these three *C. canis* isolates by different research groups. It suggests that two different genetic types of *C. canis* exist and that one divides by endodyogeny in culture and the other produces MZTC. How this would translate to *in vivo* development and pathogenicity in dogs is also an interesting area needing research.

The initial studies on the development of *C. suis* in five types of mammalian host cells in culture indicated that development was by endodyogeny (21) and stages were structurally similar to Type I meronts observed in neonatal pigs (22). Similar findings were observed in primary porcine kidney cells and primary embryonic bovine kidney cells (23). However, stages suggestive of multinucleate Type II meronts were seen in primary porcine kidney cells but no merozoites were produced (23). The ultrastructural aspects of *C. suis* development of Type I meronts by endodyogeny in these cell cultures (24) was examined and the findings were not different from those reported to occur in the small intestinal epithelial cells of infected pigs (25). Notably crystalloid bodies were seen in Type I merozoites *in vitro* (24). Complete development of *C. suis* was observed in a swine testicular cell line (26). Development was delayed 2–4 days and most stages were Type I meronts and merozoites. The ultrastructure of microgamonts, microgametes, and oocysts were described (26). Oocysts did not sporulate in culture. Studies conducted in 2013 using a porcine intestinal epithelial cell line reported similar results (27). The study demonstrated that high densities of gamonts occurred at 12 days after infection and found that an optimum infective dose was one sporozoite per 100 host cells (27).

No evidence of MZTC formation has been reported in cell cultures. Similarly, studies in pigs and potential paratenic hosts have been negative (28, 29). This is puzzling because the biology of *C. suis* conforms to all other reported features of the genus.

Four studies have examined development of sporozoites obtained from oocysts of *C. belli* in human and other mammalian cell cultures (30–33). The studies have demonstrated that development occurs in several cell types from humans including those derived from human ileocecal adenocarcinoma (HCT-8), epithelial carcinoma of the lung, and macrophages. Other mammalian cells examined have consisted of mouse macrophages, Madin-Darby bovine kidney, Rhesus monkey kidney (RMK) and African green monkey kidney (VERO) cells. Endodyogeny was described in HCT-8 cells and RMK cells using transmission electron microscopy (32). The authors found no crystalloid bodies in merozoites of *C. belli* as are present in *C. suis* (24) while undergoing endodyogeny *in vitro* but otherwise the structural findings were similar to those reported for *C. suis*. Stages of *C. belli* obtained from macrophage cell cultures were infectious for RMK cells upon subinoculation and development by endodyogeny occurred (31) but MZTC were not reported.

I have attempted to point out the similarities and important differences in the development of *Cystoisospora* species from mammals and to provide insight into future research directions. Development will occur in many host cell types but studies with *C. suis* illustrate that selection of the proper host cell type can lead to complete development thus increasing the utility of an *in vitro* system. Studies with *C. canis* suggest that genetic differences in *Cystoisospora* species exist that are reflected by the phenotype of development expressed *in vitro*. I look forward to seeing what advances in our knowledge of this group are revealed in the future.

## Author Contributions

The author confirms being the sole contributor of this work and has approved it for publication.

### Conflict of Interest Statement

The author declares that the research was conducted in the absence of any commercial or financial relationships that could be construed as a potential conflict of interest.
